# Cyanobacterial lipopolysaccharides and human health – a review

**DOI:** 10.1186/1476-069X-5-7

**Published:** 2006-03-24

**Authors:** Ian Stewart, Philip J Schluter, Glen R Shaw

**Affiliations:** 1 National Research Centre for Environmental Toxicology, University of Queensland, 39 Kessels Road, Coopers Plains, QLD 4108, Australia; 2 School of Population Health, University of Queensland, Herston Road, Herston, QLD 4006, Australia; 3 Cooperative Research Centre for Water Quality and Treatment, PMB 3, Salisbury, SA 5108, Australia; 4 Faculty of Health and Environmental Sciences, Auckland University of Technology, Private Bag 92006, Auckland 1020, New Zealand; 5 School of Public Health, Griffith University, University Drive, Meadowbrook, QLD 4131, Australia

## Abstract

Cyanobacterial lipopolysaccharide/s (LPS) are frequently cited in the cyanobacteria literature as toxins responsible for a variety of heath effects in humans, from skin rashes to gastrointestinal, respiratory and allergic reactions. The attribution of toxic properties to cyanobacterial LPS dates from the 1970s, when it was thought that lipid A, the toxic moiety of LPS, was structurally and functionally conserved across all Gram-negative bacteria. However, more recent research has shown that this is not the case, and lipid A structures are now known to be very different, expressing properties ranging from LPS agonists, through weak endotoxicity to LPS antagonists. Although cyanobacterial LPS is widely cited as a putative toxin, most of the small number of formal research reports describe cyanobacterial LPS as weakly toxic compared to LPS from the Enterobacteriaceae.

We systematically reviewed the literature on cyanobacterial LPS, and also examined the much lager body of literature relating to heterotrophic bacterial LPS and the atypical lipid A structures of some photosynthetic bacteria. While the literature on the biological activity of heterotrophic bacterial LPS is overwhelmingly large and therefore difficult to review for the purposes of exclusion, we were unable to find a convincing body of evidence to suggest that heterotrophic bacterial LPS, in the absence of other virulence factors, is responsible for acute gastrointestinal, dermatological or allergic reactions via natural exposure routes in humans.

There is a danger that initial speculation about cyanobacterial LPS may evolve into orthodoxy without basis in research findings. No cyanobacterial lipid A structures have been described and published to date, so a recommendation is made that cyanobacteriologists should not continue to attribute such a diverse range of clinical symptoms to cyanobacterial LPS without research confirmation.

## Introduction

Cyanobacterial LPS is attributed with a range of pathological effects in humans, from gastro-intestinal illness, cutaneous signs and symptoms, allergy, respiratory disease, headache and fever. This review will present the studies of cyanobacterial LPS, and will attempt to place the knowledge of these products within the broader understanding of LPS from Gram-negative heterotrophic bacteria. The paper will present an overview of the mechanisms of toxicity of Gram-negative bacterial LPS, discussing the history of its discovery and the present perception of its pathogenicity.

### Cyanobacterial LPS and symptoms in humans

Table 1 lists some of the signs and symptoms reportedly associated with exposure to cyanobacterial LPS, and references that imply particular symptoms or symptom groups are explained by such exposures. Table 1 does not present an exhaustive list of citations implicating cyanobacterial LPS with human illness. Many such references are found in review articles, and the table does not include citations which discuss cyanobacterial LPS in the context of cyanobacterial toxins without linking them to specific illnesses – e.g. "Cyanobacterial toxins are of three main types: lipopolysaccharide endotoxins, hepatotoxins, and neurotoxins." [1]; "Potential irritant; affects any exposed tissue" [2,3]; "(LPS) are responsible for the irritant nature of cyanobacterial material" [4]; and "...toxicants such as...lipopolysaccharide endotoxins...affect any exposed tissue..." [5].

**Table 1:** Signs and symptoms attributed to contact with cyanobacterial lipopolysaccharides.

**G-I**	**Skin**	**Eye**	**Allergic**	**Respiratory**	**Hay-fever**	**Headache**	**Dizziness**	**Cramps**	**Blistering of mucous membranes**	**Fever**	**References**
											[168, 169, 217, 262-268]
											[23, 269-273]
											[6, 12, 274-276]
	*										[20, 277*-279]
											[257]
			**								[11**, 280]
			***								[59, 261***]
											[26, 281]
											[17]
											[282]
											[19]
											[15, 21, 283]
											[206]
											[284]
											[285]

* "cytotoxic" skin irritation

** "allergic and toxic responses"

*** "respiratory allergy"

Several authors note that the health implications of cyanobacterial LPS are poorly understood and the topic requires more research [6-15]. Only one reference was found in the cyanobacteria literature that raises doubts about illness caused by cyanobacterial LPS: Carmichael [16] suggests that the relationship between ingested LPS and illness in an immunologically competent population is debatable, there being little evidence that people with a normal LPS-containing gut flora would be affected by LPS from water supplies.

The relationship between cyanobacterial LPS and illness is discussed in language ranging from cautious: "...may be responsible..." [17], "possibly (due to) lipopolysaccharides" [15], to definitive: "dermatotoxins" [18] and "dermatotoxic lipopolysaccharides" [19,20]. Cyanobacterial LPS has also been implicated as the cause of an outbreak of pyrogenic reactions in a haemodialysis clinic in 1974 [21].

The references in the literature to the association between cyanobacterial LPS and this rather diverse range of symptoms are not based on any research evidence specific to cyanobacterial LPS. As will be discussed later in this review, the few toxicological investigations that have been carried out to date are mostly limited to the end-points of lethality and the local Shwartzman reaction, in which sequential subcutaneous and intravenous injections of LPS produce a dermonecrotic lesion in rabbit skin. Rather, symptomatology attributed to contact with cyanobacterial LPS appears to be something of a default diagnosis for illnesses that are not otherwise explained by the current knowledge of cyanobacterial exotoxins, most of which are somewhat specific to their target organ system.

The most likely explanation for the ready attribution of these illnesses to cyanobacterial LPS lies in the realisation that LPS from Gram-negative heterotrophic bacteria are implicated in significant morbidity – and mortality – so cyanobacteria, which are widely but somewhat inaccurately accepted as Gram-negative bacteria may equally be responsible for illness because they contain LPS. Codd makes such a proposition, suggesting that: "The LPS of other bacteria are associated with gastroenteritis and inflammation problems and it is thought that cyanobacterial LPS may contribute to waterborne health incidents although this possibility has not been adequately investigated" [13].

There is a risk that speculative attribution of symptoms in humans to environmental exposure to cyanobacterial LPS will continue without an appropriate and specific research foundation. This review will attempt to examine more closely the mechanisms by which LPS from Gram-negative heterotrophic bacteria is associated with morbidity, and compare and contrast cyanobacterial LPS and heterotrophic bacterial LPS.

### Cyanobacteria: Gram-negative or Gram-positive?

Most references to cyanobacteria describe these organisms as Gram-negative prokaryotes [17,22-26]. Weckesser et al [27] state that the presence of LPS is evidence for the Gram-negative cell wall architecture of cyanobacteria. However, Weckesser et al [28] reported that a strain of *Anabaena variabilis *they investigated was Gram-positive, "like other blue-green algae...". Drews [29] also classifies cyanobacteria as Gram-positive. Golecki [30] reviewed electron microscopy studies of the cell wall architecture of various bacteria, and suggested that cyanobacteria have characteristics of both Gram-negative and Gram-positive organisms. They contain an outer membrane and LPS, which are defining characteristics of Gram-negative bacteria [31,32], and a thick, highly cross-linked peptidoglycan layer similar to Gram-positive organisms. Jürgens & Weckesser [33] and Golecki [30] suggest that the chemical and structural organisation of the cell wall may place cyanobacteria in a separate phylogenetic category to both Gram-negative and Gram-positive bacteria, with cyanobacteria developing independently of Gram-negative and Gram-positive bacteria from a common ancestor. However, according to Margulis & Schwartz [34], electron microscopy shows that cyanobacteria have Gram-negative cell walls.

### Terminology: "endotoxin" in the cyanobacteria literature

While most references to endotoxin in the cyanobacteria literature (e.g. those in Table 1) are clearly referring to LPS, i.e. cell wall structural components, this is not always the case. Several authors discuss "endotoxins" when they were obviously describing the toxic properties of microcystins [35-41]. Kay [42] refers to aphantoxin (now known as saxitoxins) from *Aphanizomenon flos-aquae *as an "alkaloid endotoxin". Gentile & Maloney [43] also labelled what were presumably saxitoxins as an endotoxin. In recent years cyanobacterial hepatotoxins and neurotoxins are still being described as endotoxins [44,45]. This misunderstanding of the nomenclature is also seen in biotoxin research fields outside of cyanobacteria, with brevetoxins described as endotoxins [46]. Immunologists clearly understand endotoxins to refer only to LPS of Gram-negative bacteria. The reader's attention is drawn to this distinction, as throughout this review "endotoxin" and "LPS" are used more or less interchangeably, whereas other cyanobacterial toxins such as microcystins, cylindrospermopsin, saxitoxins and anatoxin-a, when discussed in aggregate are referred to as "exotoxins". The history of the term "endotoxin" and its current use are discussed in the following section.

### Gram-negative bacterial LPS: introduction and its discovery

LPS from many bacterial species will initiate acute inflammatory responses in mammals that are typical of the host reaction to tissue injury or infection [47]. LPS can induce a large and diverse range of effects, ranging from pyrexia to Gram-negative septic shock, which manifests as a complex and dramatic syndrome involving fever, leucopoenia, hypotension, cardiopulmonary dysfunction, disseminated intravascular coagulation and multi-system failure [48,49].

The history of the understanding of LPS starts in the late nineteenth century, when Richard Pfeiffer used heat-inactivated lysates of *Vibrio cholerae *to provoke pathophysiological effects in guinea pigs [48]. Pfeiffer named the heat-stable and cell-associated toxic substance "endotoxin", to distinguish it from the heat-labile and proteinaceous exotoxins, which were known to be secreted by replicating bacteria [48-51]. Initial analyses of endotoxin revealed that it contained polysaccharide and lipid, and was thus named lipopolysaccharide. The terms "endotoxin" and "lipopolysaccharide" are widely used by workers in a variety of biomedical fields as synonyms to describe the same molecule (many references, e.g. [48,51-57] including the field of cyanobacteria research: "lipopolysaccharide endotoxins" [20,58-60]. However, some authors have suggested that LPS should refer to the purified molecule, whereas endotoxin more appropriately describes macromolecular complexes of LPS, protein, phospholipid and nucleic acids [49,50,61].

The study of LPS, which was largely concerned with investigating fever, progressed in the late 1940s with the discovery of endogenous pyrogens [49]. Since then, the field of cytokine biology has made enormous strides. It is now understood that the pathological effects of LPS are indirect, i.e. LPS acts by initiating a cascade of host-mediated responses: initially monocytes and macrophages are stimulated, and then neutrophils and platelets congregate in microcapillaries, causing vascular injury. Inflammatory cells release a range of endogenous mediators, including arachidonic acid metabolites, platelet-activating factor, cytokines such as interleukin-1 (IL-1), IL-6 and tumour necrosis factor-alpha (TNF-α), nitric oxide, toxic O_2 _metabolites, vasoactive amines, proteases and products of the complement and coagulation cascades. There are many reviews in the literature on cytokines and LPS – some recent examples are: [47-49,51,55,56,62-64]. Cytokine-mediated responses are extremely complex: cytokines are pleiotropic, i.e. a single type of cytokine affects several different types of cell. Cytokines have autocrine effects, i.e. they can stimulate the cells that secrete them to produce more cytokines, and paracrine effects – stimulating other cells to produce different cytokines [65]. Signs and symptoms in human volunteers after administration of endotoxin are: fever, rigors, and influenza-like symptoms of fatigue, headache, nausea, myalgia, arthralgia, drowsiness, mild amnesia and diarrhoea [47,61,66,67].

From the discovery that LPS-associated pathology results from the stimulation of host cell responses came the realisation that LPS binds to specific receptors in order to elicit the release of cytokines and other inflammatory mediators [64]. Several membrane-bound and soluble proteins have been shown to bind LPS; the most important appear to be CD14 and LPS-binding protein (LBP) [48,51,52,55,63,64,68]. The Toll-like receptor (TLR) family are a recently discovered group of transmembrane receptors that are fundamental in signalling innate immune responses to conserved microbial structures, and they are also involved in the recognition of some endogenous ligands. TLR4 is instrumental in signalling LPS from many Gram-negative bacteria, and TLR2 is involved in the recognition of some unusual LPS. There is a considerable and rapidly expanding body of literature on the TLR family; one of the most comprehensive reviews is by Takeda et al [69]. A review by Janeway and Medzhitov [70] is an excellent introduction to TLRs, and places the activity of these receptors in the broader context of innate immunity.

Knowledge of the role of specific receptors in mammalian hosts has led to the demonstration that LPS itself is non-toxic. Schnaitman notes that "LPS itself is not toxic" [71] and Henderson et al describe LPS as "relatively inactive" and observe that several host proteins are necessary for LPS to display full agonist potency [49]. CD14-deficient cell lines such as Chinese hamster ovary are unresponsive to LPS at high doses, and cell lines that show poor LPS activation can be converted to show high activation when transfected with the CD14 gene [71]. LPS-unresponsive mouse strains and cytokine knockout strains also serve to reinforce the concept that LPS is not directly toxic, and the pathophysiology associated with Gram-negative LPS results from host-mediated factors [68,72-77].

### LPS structure – similarities and differences across Gram-negative bacteria

**Figure 1:**
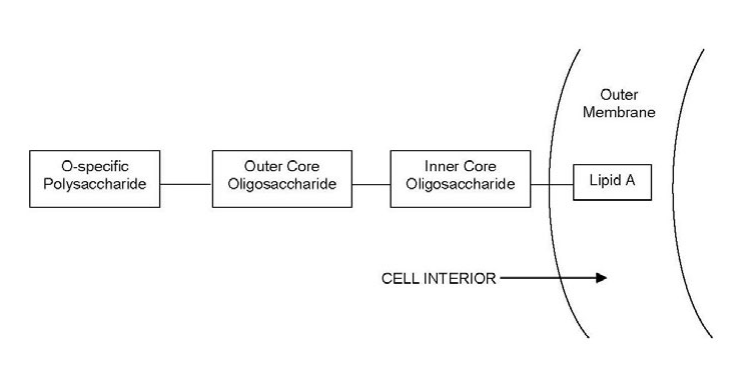
**Schematic of the basic LPS structure**. The O-specific polysaccharide is the unit that is most exposed to the external environment and so manifests the greatest structural diversity; lipid A is the most conserved structure.

LPS from different Gram-negative species apparently share common features in their basic architecture. A structure consisting of four covalently linked segments – a surface carbohydrate polymer (O-specific chain), a core oligosaccharide featuring an outer and inner region, and an acylated glycolipid (termed lipid A) – is seen in such ecologically diverse bacteria as *Salmonella, Pseudomonas, Vibrio *and *Rhizobium *[78]. The O-specific chain shows the most diversity, and is the basis for serological specificity [48], while lipid A, which anchors the LPS molecule in the Gram-negative outer membrane, is the most conserved biochemical structure across different bacterial species [72] (See Figure 1). There is unequivocal acceptance that the lipid A moiety is the innate immune stimulating or "endotoxic" component of LPS [63,79]. This was confirmed by the observations that LPS from polysaccharide-deficient mutant strains were equally as bioactive as parent LPS [63,72,80], and chemically synthesised *Escherichia coli *lipid A exhibits identical activity to natural *E. coli *lipid A [63,81].

**Figure 2:**
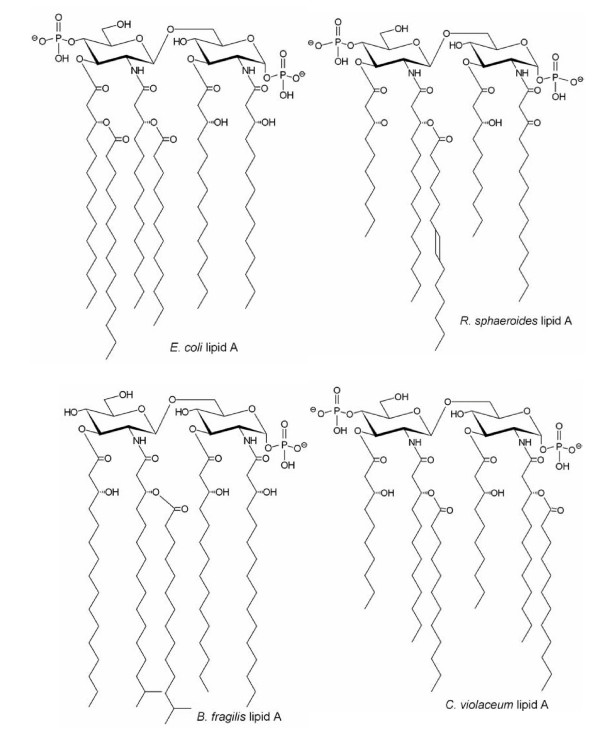
**Primary lipid A structures**. *E. coli *has a bis-phosphorylated diglucosamine backbone with six amide and ester linked fatty acyl chains. The non-endotoxic (and LPS-antagonist) *R. sphaeroides *lipid A has an identical disaccharide backbone but has five acyl residues, shorter ester-linked primary acyl chains and an unsaturated acyl group. *B. fragilis*, which expresses low endotoxic potential, has a mono-phosphorylated backbone and five acyl chains with longer chain lengths than those seen in *E. coli*. Lipid A from *Chromobacterium violaceum*, like *R. sphaeroides*, is an LPS antagonist. Figures adapted from: Weintraub et al [91], Rietschel et al [78] and Takayama & Qureshi [84].

### Are all LPS equal in terms of their host-mediated responses?

Although the basic structure of lipid A is seen in phylogenetically diverse Gram-negative bacteria, variations in the nature and location of acyl groups or alterations in the hydrophilic backbone can result in partial or total loss of biological activity [48,51,63,82,83]. An example is seen in the case of some purple non-sulfur bacteria, which could be considered to be somewhat more representative of cyanobacteria when it comes to drawing comparisons on the biological activity of LPS. Purple non-sulfur bacteria, being photosynthetic, are Gram-negative bacteria that occupy a similar ecological niche to that of cyanobacteria, and therefore have growth and reproductive strategies that much more closely resemble cyanobacteria than the heterotrophic, gut-dwelling *E. coli *and *Salmonella*. Lipid A complexes from *Rhodobacter sphaeroides *and *Rhodobacter capsulatus *are not only inactive, but are antagonists of Enterobacteriaceae LPS-induced cell activation [48,49,52,63,84]. *R. sphaeroides *and *R. capsulatus *lipid A differs from that of *Escherichia coli *in several respects, all relating to the acylation pattern; the lack of endotoxic activity in *R. sphaeroides *and *R. capsulatus *appears to be attributable to the presence of five rather than six fatty acid groups, and the shorter chain lengths (C_10 _in *R. sphaeroides *and *R. capsulatus *compared with C_14 _in *E. coli*) in two ester-linked fatty acids [84]. Lipid A from *R. sphaeroides *and *R. capsulatus *is of pharmacological interest because of their LPS-antagonist properties; these lipid A structures and some synthetic derivatives have been shown to be potent LPS antagonists *in vitro *and to protect against LPS-induced morbidity and mortality in animal models [49,63,78,85]. Competitive inhibition of biologically active LPS by LPS antagonists further illuminates the requirement for host cell receptor sites in mediating the responses to LPS [52]. Other purple non-sulfur bacteria, e.g. *Rhodopseudomonas viridis *and *Rhodopseudomonas palustris *are also reported to lack endotoxic activity [27,84], but the lipid A from another purple non-sulfur bacterium, *Rubrivivax gelatinosus*, is reportedly associated with high lethality in mice – at similar doses that cause lethality in *Salmonella *– and high pyrogenicity in rabbits [27,84]. The high endotoxicity of *R. gelatinosus *is apparently due to the presence of six fatty acid groups in the lipid A structure [84,86]. Lipid A complexes from the pathogenic bacteria *Chlamydia trachomatis *and *Legionella pneumophila *and the non-pathogenic hyperthermophile *Aquifex pyrophilus *reportedly possess little or no LPS agonist or antagonist properties [63].

*Bacteroides *spp., which are common gut and periodontal commensals, have low endotoxic activity compared to that of *Salmonella*, with a greater than 500-fold higher mouse LD_50 _and 100 to 1,000-fold reduced ability to stimulate production of IL-1 in monocyte cultures [87,88]. *Bacteroides fragilis *LPS inhibits *E. coli *LPS-induced endothelial adhesiveness for polymorphonuclear leucocytes, although *B. fragilis *LPS is reported to be directly toxic to endothelial cell cultures at high concentrations [52]. In a study of cytokine induction in whole blood, Frieling et al [89] showed that *B. fragilis *stimulated IL-1β, IL-6 and IL-1ra at levels comparable to Gram-positive bacteria, where 100 to 1,000-fold more organisms are required to produce similar concentrations of cytokines than with common pathogenic Gram-negative bacteria. Wilson et al [90] suggest that *B. fragilis *may be such a weak cytokine inducer because, being a dominant organism in normal gut flora, it has evolved mechanisms to downregulate the synthesis of inflammatory cytokines in order to optimise their niche in the host gut. *B. fragilis *has five fatty acyl groups in the lipid A moiety, and other structural differences to *Salmonella *and *E. coli *LPS, such as a monophosphorylated disaccharide backbone and longer fatty acyl chains [91].

Some pathogenic bacteria have LPS which reflects the highly specific niches they inhabit: enteric Gram-negative bacteria have long, hydrophilic and neutral O-specific polysaccharide chains which protects the organism from solubilisation by bile acids and intestinal enzymes, whereas organisms that colonise the mucous membranes of the respiratory and genital tracts have outer membrane surfaces that are hydrophobic and can be solubilised by bile [92,93]. Gram-negative bacteria such as *Neisseria, Haemophilus *and *Bordetella *have developed unique surface glycolipids lacking O-antigens, which some workers call lipooligosaccharides [63,92,93].

The presence of one or more secondary acyl chains appears to be essential for lipid A to stimulate some endotoxic reactions [82,88]. One of the final stages of Enterobacteriaceae lipid A biosynthesis is the formation of acyloxyacyl groups, so-called secondary fatty acids [94]. A leucocyte enzyme, acyloxyacyl hydrolase, selectively removes these secondary fatty acyl groups, without releasing the 3-hydroxy acyl chains that substitute the lipid A disaccharide backbone [82,95]. Deacylated LPS from *E. coli, Salmonella typhimurium, Haemophilus influenzae *and *Neisseria meningitidis *were shown to have reduced activities in a series of tests relating to endotoxic potential, in some cases by greater than two orders of magnitude, and deacylated *Neisseria *LPS demonstrated some antagonistic activity towards *Neisseria *and *Salmonella *LPS [82,95,96].

The lipid A analog, compound 406, which lacks the two secondary fatty acids of *E. coli *lipid A, is unable to induce cytokines in human cells [48,51,75]. Rietschel et al [51] propose that endotoxic capacity resides in the spatial conformation of lipid A, in that biologically inactive lipid A (e.g. *R. capsulatus*) conforms to lamellar structures, whereas endotoxic lipid A adopts exclusively cubic or hexagonal structures.

Netea et al [97] suggest that the historical assumption that LPS from different Gram-negative bacteria possess similar biological effects is incorrect, and that differences between LPS across species are the rule rather than the exception. The authors discuss structure-function relationships between LPS and the Toll-like receptors (TLRs), which are integral in LPS-mediated signalling, suggesting that differences in the three-dimensional conformation of LPS molecules translate into differences in TLR signalling of proinflammatory cytokines. In their reviews of the supramolecular structure and phase transition states of LPS and lipid A, Seydel and Brandenburg [98] and Seydel et al [54] suggest that endotoxic activity is related to the three-dimensional conformation of LPS and the multimeric aggregates they form. The conformation of LPS or lipid A within such aggregates is not constant, but a reversible phase transition occurs, which is temperature-dependent and related to the length and degree of saturation of the acyl chains and other physiological conditions.

More recent work by Seydel and collaborators has led to the realisation that the biological activity of specific lipid A structures can be determined by an understanding of their supramolecular structure, which is a function of the monomeric conformation, which in turn is largely determined by the primary molecular structure [83,99,100]. The presence of sufficient negative charges on the disaccharide backbone – mainly, but not necessarily, two phosphate groups – has an important influence on lipid A molecular conformation and the binding capacity to serum proteins such as LBP [83,101]. A high negative charge density is reported to be an essential requirement for agonistic and antagonistic properties; complete or partial substitution of negative charges can result in the loss of all biological activity [83,102]. Seydel's team now suggest that a general principle can be applied regarding the molecular conformation of lipid A structures and their biological activity: lipid A structures that adopt conical/concave shapes have hexagonal or cubic supramolecular aggregate structures and express high endotoxic potential, whereas lipid A structures that adopt cylindrical conformations have lamellar aggregates and are either inactive or LPS antagonists [99]. Thus lipid A of the photosynthetic bacterium *R. gelatinosus*, which has high endotoxic activity [27,84], was seen to adopt hexagonal aggregate structures, whereas the lipid A formations of other non-endotoxic photosynthetic bacteria adopt cylindrical shapes and lamellar aggregate structures [99,100,103]. Lipid A from *Chromobacterium violaceum *forms a cylindrical geometry and is reportedly three orders of magnitude less active than *E. coli *lipid A [104]. Ulmer et al [105] also found that a synthetic lipid A structure based on *C. violaceum *lipid A had markedly lower cytokine-inducing capacity than an *E. coli*-based synthetic lipid A. These findings are in contrast to some other reports, which describe *C. violaceum *lipid A as having a high agonist activity [51,75]. Of interest is the observation that the primary lipid A structures of *C. violaceum *and *R. gelatinosus *are very similar (bisphosphorylated diglucosamine backbone, six symmetrically distributed acyl chains differing only in the length of the two acyloxyacyl groups: C = 12 in *C. violaceum *and C = 10 for *R. gelatinosus*) [84]. Yet *C. violaceum *lipid A is an LPS antagonist, whereas *R. gelatinosus *lipid A expresses high agonist activity. Seydel et al[104] suggest that the chemical structures of these two lipid A complexes may need to be re-examined. Lipid A of *Campylobacter jejuni*, which has low endotoxic potential, shows a very slight tendency to adopt a conical/concave shape, whereas the lipid A of *E. coli *clearly adopts a conical/concave form [99,106].

As a result of these studies, the ability of a lipid A monomer to adopt a conical shape (the so-called endotoxic conformation) has been described as a prerequisite for endotoxicity [100]. Seydel et al [104] suggest that when lipid A molecules are intercalated into target cell membranes, only lipid A which forms a conical shape – where the cross-sectional area of the hydrophilic backbone is smaller than the cross section of the hydrophobic acyl groups – can exert a mechanical stress on signalling proteins. LPS with a lipid A moiety which assumes a cylindrical shape – cross-sectional areas of the hydrophobic and hydrophilic components being roughly equal – will occupy the binding site but be unable to activate signalling proteins, thus acting as an LPS antagonist [102,104]. The number and distribution of acyl chains has been shown to affect the tilt angle of the disaccharide backbone with respect to the target cell membrane; the orientation of the backbone sugars appears to correlate with the endotoxic potential of the LPS. The lipid A molecular shape and the tilt angle of the backbone sugars are reported to be the complete determinants of endotoxic activity [102,104].

The discussion above has contrasted the endotoxic potential of lipid A structures from the most widely studied forms, those of the Enterobacteriaceae, with some unusual lipid A complexes from photosynthetic bacteria and synthetic lipid A analogs. The reason that cyanobacterial LPS has not been discussed here is simply that the required research has not been done as yet. No cyanobacterial lipid A structures have been published, therefore no inferences can be deduced as to their likely endotoxic potential, or lack of it. But with the knowledge that endotoxic potential can vary in the most fundamental way across Gram-negative bacteria, from agonistic to weakly active to inactive to antagonistic, it should be incumbent on the cyanobacteria research community to cease attributing biological activity and clinical symptoms to cyanobacterial LPS without specific research evidence. Cyanobacteria may not be typical Gram-negative organisms because of their unusual cell wall architecture, and cyanobacteria will have experienced very different selection pressures to gut-dwelling Gram-negative bacteria, which may be reflected in different lipid A structures.

### LPS and infection

Much research into LPS and lipid A has understandably concentrated on the severe, life-threatening host responses to circulating LPS that constitute Gram-negative septicaemia and septic shock. Many experimental models have utilised either *in vitro *studies of isolated cell lines or animal and human studies where LPS is exposed to the circulation by parenteral injection.

The matter of the degree to which cyanobacterial LPS/lipid A can stimulate mammalian cytokine networks under experimental conditions, which remains largely unanswered, is only one aspect in the understanding of cyanobacterial LPS and the gastro-intestinal, respiratory and cutaneous illnesses which have been attributed to contact with it. The other side of the story that needs to be considered is the mechanism by which cyanobacterial LPS might (and might not) stimulate endotoxic responses by the various natural exposure routes: ingestion, inhalation and contact. The following discussion will briefly review the association between acute gastrointestinal illness and Gram-negative bacterial LPS. Cutaneous responses to LPS will be briefly discussed.

### Terminology: infection, pathogen

Infectious diseases caused by bacteria are characterised by several discrete steps: bacterial adhesion to the host, colonisation within or on the host, and evasion of host defences [31,49]. The only references in the literature that describe cyanobacteria as invasive, infectious organisms were from two authors:

• Rank [107-109] put forward the hypothesis that a chronic, low-virulence infestation by cyanobacteria growing heterotrophically may explain the aetiology of arteriosclerosis in humans and homeothermic animals. The author bases his theory on ecological data, examining the geographical, demographic and historical distribution of the disease. Routes of infection are posited to be ingestion of unfermented milk, public water supplies drawn from surface waters as distinct from groundwater [108,109], and earth-contaminated food and other objects [107]. The author also critiques the three theories of the pathogenesis of arteriosclerosis that were current at the time (response-to-injury, lipid hypothesis, and monoclonal hypothesis) and suggests laboratory studies to replicate and study the disease. Such studies do not subsequently appear in the literature, and no further reference to the cyanobacterial infection hypothesis can be found.

• Ahluwalia et al [110] and Ahluwalia [111,112] posited the theory that *Microcystis aeruginosa *is the causative organism of the mostly tropical, water-exposure-related invasive disease rhinosporidiosis. However, this theory has been disputed; convincing and probably conclusive evidence appears to place the eukaryotic protist *Rhinospiridium seeberi *as the causative organism (see Author's reply to Ahluwalia [112]).

Most public health workers would presumably place cyanobacteria-related illnesses in the context of environmental exposures, as distinct from familiar diseases due to communicable infectious bacteria, which in many cases feature transmissible illnesses, secondary to the original reservoir of infection. Cyanobacteria-related illness is viewed as intoxication, rather than infection, usually on the basis of a sudden onset of symptoms occurring soon after exposure (i.e. without an incubation period), and lack of secondary cases [113]. Giesecke [114] defines infectious disease as "all diseases caused by micro-organisms", with sub-definitions of communicable and transmissible disease (communicable disease: capable of being transmitted from an infected individual to another person, directly or indirectly; transmissible disease: able to be transmitted from one individual to another by 'unnatural' routes). Whether cyanobacteriologists would embrace that definition of infectious disease is debatable, but most would agree that cyanobacteria-related diseases are neither communicable nor transmissible. Exotoxin-producing cyanobacteria certainly fit the dictionary definition of pathogenic (i.e. disease-causing) organisms; the reader's attention is drawn to the distinction between infectious (implying colonisation and evasion of host defences) Gram-negative bacteria and non-infectious cyanobacteria in the following discussions on LPS and G-I and dermal illnesses.

### Mechanisms of vomiting and its relationship to LPS activity

Nausea and vomiting are normal physiological responses to the ingestion of toxic substances; they are essential defences because they are the end result of the actions of sensorimotor systems that operate to identify and rapidly expel hazardous substances from the upper G-I tract [115-117]. The two main sensory systems that direct the emetic response are local, associated with the gut mucosa (pre-absorptive response), and central, specifically the chemoreceptive trigger zone of the area postrema, located in the dorsal surface of the medulla oblongata (post-absorptive response) [118-120]. Stimulation of chemoreceptors in the stomach, jejunum and ileum by irritant chemicals such as hypertonic saline, copper sulfate or mustard, or by bacterial enterotoxins, leads to the activation of vagal sensory afferent nerves to the brain. Vagal efferent processing through the enteric nervous system stimulates enteric motor neurons to effect emesis [115,117,119,120]. Emetic chemoreceptors are also found in the vascular system; activation of these chemoreceptors will also initiate nausea and vomiting [115]. Endogenous mediators of emesis such as dopamine, acetylcholine and enkephalin are reported [115]. Prostaglandins have well-known emetic actions [121].

Circulating *E. coli *LPS is a potent emetic stimulant. In a series of experiments using piglets, Girod et al [122] showed that parenteral administration of LPS provoked vomiting (as well as fever, rigors, purpura, diarrhoea and drowsiness). The authors suggested that LPS stimulates vomiting by means of cytokine-induced prostaglandins and other endogenous mediators acting both centrally and on vagal afferents. Other animal studies have demonstrated the emetic action of LPS [123].

The area postrema is reported to be the primary sensory area involved in nausea as well as vomiting, although nausea is accompanied by autonomic excitation, whereas vomiting is a somatic process independent of the autonomic nervous system [118,121]. Presumably LPS-stimulated endogenous mediators are associated with symptoms of nausea, which is reported in several studies and reviews of intravenous exposure to LPS in human volunteers [47,61,66,67].

To investigate vomiting associated with exposure to cyanobacteria, the appropriate research efforts should determine whether cyanobacterial LPS is capable of stimulating gut chemoreceptors, or if cyanobacterial LPS can gain access to the circulation and stimulate nausea and vomiting centrally. Another point of interest should be directed towards the cyanobacterial exotoxins, as to their capacity to stimulate either gut mucosal chemoreceptors, vascular emetic chemoreceptors, or whether they induce vomiting through the activity of endogenous mediators. Bacterial exotoxins such as staphylococcal enterotoxins (SEs) are known to stimulate emesis via gut chemoreceptors [116,117,124]. Worthy of note is that experimental induction of LPS-related emesis is achieved by intravenous or intraperitoneal routes [122-127], whereas SEs readily elicit vomiting when administered intragastrically [124,128]. Also of interest with respect to cyanobacteria-related G-I illness are some similarities of clinical symptoms in the case of staphylococcal food poisoning: enteritis due to ingestion of SEs is characterised by rapid onset (1–4 hours) of vomiting ± nausea and diarrhoea, abdominal cramping and dizziness [124,129]. Staphylococcal food poisoning is an intoxication, not an infectious process [129].

### Diarrhoea and LPS

One inference that can be drawn from the references that posit cyanobacterial LPS as the cause of G-I illnesses (see Table 1) is that these symptoms are not necessarily related to exposure to any of the known cyanobacterial exotoxins, the assumption being that exposure to – and presumably illness caused by – cyanobacterial LPS can occur with or without concurrent exposure to exotoxins (i.e. from non-toxic strains or species, or non-production of exotoxins at the time of exposure). However, several questions need to be answered if LPS is the sole presumptive "G-I-toxin". A hypothesis is needed for the mechanism of cyanobacterial LPS, in the absence of other virulence factors, to initiate diarrhoea by the oral exposure route. Another explanation for cyanobacterial LPS-related diarrhoea is that the alteration in gut membrane permeability may be related to a cytokine cascade generated by circulating cyanobacterial LPS, but again, a hypothesis is needed to explain the exposure route for the LPS to overcome innate immune intestinal defences in order to gain access to the circulation.

Some observations on the behaviour of Gram-negative bacterial LPS in the gut serve to cast doubt on the suspicions that cyanobacterial LPS alone is responsible for initiating acute gastro-intestinal illness in humans by the oral route:

• **Commensal gut flora: **The human intestinal tract houses an enormous population of bacteria, many of which are Gram-negative. The Enterobacteriaceae are found in normal faecal flora at some 10^8^–10^9 ^per gram [130]. The number of microbes in the gut lumen exceeds the number of eukaryotic cells in the human body by an order of magnitude [49,131], an observation that may lead some to unkindly suggest that the principal reason for human existence is to serve as bags for the housing and transport of bacteria. Nanthakumar et al [132] note that mature enterocytes are 100 to 1,000 times less sensitive to LPS than neutrophils and hepatocytes, which is not surprising since they are exposed to Gram-negative bacteria and their endotoxins since birth when the gut is colonised.

• **Non-virulent strains: **Most Gram-negative organisms are non-pathogenic. Pathogenicity involves a complex interaction between host-related and specific microbial virulence factors – the latter including pili, fimbriae and heat shock proteins [133,134]. Infectious, i.e. colonising, microbes are the most common cause of diarrhoea worldwide; pathogenic strains commonly cause disease by the action of enterotoxins [135]. That virulence factors other than lipid A structures of LPS are responsible for gastro-intestinal disease is seen in the protective effects of attenuated or mutant Gram-negative bacteria when used as live oral vaccines against pathogenic strains [133,136-138]. Some *E. coli *strains are used as probiotics for the treatment of gastrointestinal disease and infection prophylaxis in neonates [139].

• **Anecdotal reports of consumption of non-hazardous cyanobacteria: **Heaney [39] reports observations of cattle seen drinking from two Irish lakes affected by thick scums of *Anabaena flos-aquae *and *Aphanizomenon flos-aquae *without ill effect. Author IS can add a similar observation: during recruitment for an epidemiology study [140] at Lake Coolmunda in southern Queensland, a frank *Microcystis aeruginosa *bloom was in attendance. A group of six or seven dogs were seen playing vigorously in the water, and three dogs were observed drinking from it. The owners of the animals were questioned the following day; all denied observing any adverse effects. The consumption of *Spirulina *and other cyanobacteria provides further evidence that cyanobacterial LPS cannot all be harmful. Cyanobacteria as food, medicine and livestock feed will be discussed later in this review.

Oketani et al [141] state that orally administered LPS is not harmful to animals, which is in stark contrast to LPS administered parenterally. Evidence for harmful effects of orally administered LPS is difficult to gather from the literature because of the overwhelming number of publications describing experimental use of LPS as an *in vitro *and *in vivo *immune stimulant. We found two reports of *in vivo *LPS activity by the oral route: Yang et al [142] showed that oral administration of *E. coli *LPS enhanced the progression of hepatoma in rats treated with thioacetamide. Yoshino et al [143], using a mouse model of autoimmune disease, demonstrated that orally-dosed LPS exacerbated collagen-induced arthritis. These models of liver disease and autoimmunity are not applicable to the concept of acute G-I symptoms caused by cyanobacterial LPS in presumably healthy people in recreational settings and through cyanobacterial contamination of drinking water supplies. However, they highlight the importance of gut mucosal immunity. LPS and other microbial products are constantly sampled by gut-associated lymphoid tissues, which contain the largest assemblage of immunocompetent cells in the body [131]. Translocation of small amounts of LPS from the gut lumen across the epithelium and into the portal circulation is an important immune-stimulating process, with Kupffer cells in the liver playing an important role in clearance of LPS from the circulation [131]. LPS and other bacterial products from the normal gut flora can be a source of infection and sepsis when the integrity of the gut mucosa is disrupted. This occurs in a variety of disease states, including hypovolaemic shock, burn injury, trauma, acute liver failure, pancreatitis, cirrhosis and inflammatory bowel disease [131].

Roth et al [144] suggest that endogenous (i.e. gut-derived) LPS is a potent synergist of the toxicity of a range of structurally and functionally unrelated hepatotoxic xenobiotic agents. The authors put the proposition that hepatotoxicity associated with some chemicals is indirectly caused by primary damage to the intestinal tract, which allows increased translocation of bacterial LPS into the portal circulation. The liver is then exposed to harmful levels of LPS, and the ensuing liver injury resembles that caused by large doses of LPS: changes in sinusoidal and parenchymal cells, neutrophil and platelet accumulation in sinusoids, then multifocal hepatocellular degeneration and necrosis. Yee et al[145] showed that co-administration of individually non-toxic doses of LPS and the alkaloid phytotoxin monocrotaline produced significant liver injury, characterised by midzonal and centrilobular apoptotic and necrotic changes, coagulation and congestion, and loss of sinusoidal architecture. Ganey & Roth [146] propose that the activation and increased expression of various soluble mediators, signalling molecules and cellular processes are crucial events in the augmentation of xenobiotic toxicity by bacterial LPS, and that the mechanisms of toxicity are complex and variable.

Purified microcystins as well as microcystin-producing and cylindrospermopsin-producing cyanobacteria have been shown in accidental poisonings and through *in vivo *and *in vitro *experimental work to damage the gastrointestinal tract [37,147-153]. The possibility should be considered that cyanotoxins and LPS (from cyanobacteria and/or from gut-dwelling heterotrophic bacteria) may cross a disrupted gut mucosal barrier and enhance the pathological effects of cyanobacterial exotoxins.

### LPS and cutaneous reactions

As with LPS and oral exposure, searching bibliographic databases for evidence of LPS/endotoxin as the primary cause of acute clinical dermatoses is a difficult task, again because of the many citations of *in vitro *immunology work using LPS to investigate various dermal cell processes. As a starting point, dermatology textbooks were perused, yet only one of a dozen or so standard texts made any reference to either LPS or endotoxin: Rietschel & Fowler [154] describe a single case report of a hospital worker who suffered dyshidrosis (a vesicular or vesicopustular eruption on the palms of the hands), which was linked to endotoxin in latex gloves. Various dermatoses are clearly associated with either superficial or systemic infection by many Gram-negative organisms, most notably *Pseudomonas aeruginosa *[155]. However, it is unreasonable to compare mechanisms of cutaneous disease from colonising Gram-negative bacteria to those due to cyanobacteria solely on the basis that both organisms contain LPS.

### Cyanobacterial LPS

Literature searches using PubMed and Web of Science with the search terms (cyanobacteria* OR blue green alga*) AND (LPS OR endotoxin) revealed 17 publications that describe the extraction ± purification of cyanobacterial LPS. The Westphal hot phenol/water method was used in 15 of these studies (described in [156]). Jürgens et al [157] used a sucrose density centrifugation and Triton X-100 extraction, and Papageorgiou et al [158] compared the phenol/water method with novel extraction methods. Raziuddin et al [159] used chloroform and acetic acid extraction to isolate lipid A from their LPS extract. Table 2 lists the studies in which cyanobacterial LPS were tested for lethality in mice. All doses were reported in the original papers in terms of dose per mouse; these doses have been converted to mg/kg body weight for comparison purposes in Table 2. An assumed weight of 20 g was applied to mice for the studies in which the authors did not report the weight of their animals. Note that the study of Scholtissek et al [160] used galactosamine-sensitised mice, so their LPS doses are not directly comparable to those given in the other studies; a similar situation applies with the study of Katz et al [161], where adrenalectomised mice were used. Adrenalectomy sensitises mice by three orders of magnitude to the lethal effects of LPS [162,163]. By way of comparison, some examples of reported lethal concentrations of Enterobacteriaceae LPS are: 5–20 mg/kg [164], 6 mg/kg [165] and 24 mg/kg [163] (LD_50 _concentrations in various strains of LPS-sensitive mice; assumed weight of mice = 20 g) to LD_80 _concentrations of 10–23 mg/kg [166].

**Table 2:** Cyanobacterial lipopolysaccharides and lethality

**CYANOBACTERIUM**	**LETHALITY**	**REFERENCE**
*Anacystis nidulans*	non-toxic at 10 mg/kg**	[172]
*A. nidulans *KM	2.5 mg/kg^† ^(= approx. 800-fold greater than *Salmonella minnesota *LPS)	[161]
*Phormidium *spp. (x3)	all non-toxic at mean dose of 333 mg/kg	[286]
*Schizothrix calcicola*	non-toxic at 200 mg/kg	[168]
*Anabaena flos-aquae *UTEX 1444 *Anabaena cylindrica *UTEX 1611 *Oscillatoria brevis*	non-toxic at 250 mg/kg LD_50 _130 mg/kg LD_50 _190 mg/kg	[169] [169] [169]
*Microcystis aeruginosa *006 lipid A *M. aeruginosa *NRC-1 lipid A	LD_50 _approx 45 mg/kg** LD_50 _60 mg/kg** LD_50 _40 mg/kg** LD_50 _approx 45 mg/kg**	[159] [159] [159] [159]
*Spirulina platensis *Lb 1475/4a	LD_100 _425 mg/kg**	[223]
*Microcystis *sp. PCC 7806	1 of 3 mice* died at 50 μg/kg**	[160]
*M. aeruginosa *(bloom sample) *Cylindrospermopsis raciborskii *AWT 205	no deaths at 70 mg/kg no deaths at 70 mg/kg	[156] [156]

*galactosamine-sensitised (= TNF-α hypersensitised)

^† ^adrenalectomised mice

** assumed weight of mice: 20 g

In addition to lethality, various other toxicity endpoints were examined in some of the studies listed in Table 2 and others. Buttke & Ingram [167], Keleti et al [168] and Keleti & Sykora [169] investigated the local Shwartzman reaction, which is a dermonecrotic lesion elicited in rabbits by subcutaneous preparative and intravenous provocation injections. Positive Shwartzman reactions were generated by *Agmenellum quadruplicatum *LPS, *Schizothrix calcicola *LPS and *Anabaena flos-aquae *LPS, but not by *Oscillatoria tenuis *LPS [167-169]. These results should be regarded as qualitative, as no reports were made of quantitative measurements of lesions, which can be done by various methods [170,171]. However, the local Shwartzman response to *A. quadruplicatum *LPS led the study authors to describe *Agmenellum *LPS as "a less effective endotoxin than *Salmonella *LPS" [167]. The Shwartzman reaction to *A. flos-aquae *LPS was reportedly "weakly positive" [169]. Keleti & Sykora [169] also used a rabbit isolated ileal loop assay, which did not show any positive findings from the three cyanobacterial LPS isolates injected. Weise et al [172] tested *A. nidulans *LPS for pyrogenicity in rabbits, reporting a tenfold lower response than that seen from *E. coli *LPS. Schmidt et al [173] also tested the LPS from two *Synechococcus *strains for pyrogenicity, with maximum increases in rabbit body temperature of 1.5°C after injection of up to 1 mg/kg. The authors reportthat these doses are some three orders of magnitude higher than those required of *Salmonella *LPS to achieve the same effect. LPS extracted and purified by author IS [156] and also by Papageorgiou et al [158] were investigated for their potential to affect thermoregulation in a mouse model. LPS from several cyanobacterial isolates induced sickness behaviour and transient hypothermia at doses of 70 mg/kg i.p.; similar responses in positive control animals were seen after i.p. injection of 4 mg/kg *E. coli *LPS. Cyanobacterial LPS doses of 7 mg/kg also initiated transient hypothermia and sickness behaviour, although at markedly lower intensity and duration than was seen with *E. coli *LPS at 4 mg/kg [156].

Most workers concluded that the cyanobacterial LPS they examined were weakly toxic when compared to the activity of positive control heterotrophic bacterial LPS. The exception was the work of Best et al [174], who investigated the potential of isolated cyanobacterial LPS to reduce the activity of glutathione *S*-transferases (GSTs) in Zebra fish embryos. They reported that cyanobacterial LPS reduced microsomal and soluble GSTs *in vivo *to a greater extent than LPS from *E. coli *or *Salmonella typhimurium*. The authors suggested that such reduction in GST availability may deleteriously affect the ability of organisms to detoxify microcystins, presumably through decreased utilisation of glutathione (GSH) for conjugation reactions. This would seem to be a reasonable assumption, although other interpretations of this finding are possible. While the GSH system is an essential intracellular redox buffer, preventing oxidative injury, it is also an important participant in many cellular functions, including DNA and protein synthesis, metabolism, cell growth and amino acid transport [175,176]. Many enzymes are GSH-dependent, including glutathione transferases [177]. GSH is also an essential component of some immune functions such as apoptosis, T-lymphocyte signalling and proliferation, and activation of the nuclear transcription factor NFκB [176,178-184]. It is these immunological functions of the GSH system that present as somewhat paradoxical: glutathione depletion has a protective effect on various models of apoptotic and necrotic liver injury [181,185-187]. Glutathione depletion has also been shown to prevent LPS-induced inflammatory lung injury by attenuating neutrophil activation and sequestration [188,189]. Glutathione depletion has been proposed as a novel anti-inflammatory pharmacotherapy [186,190]. Dröge et al wrote in 1994 that:

"It is clear that GSH is one of the limiting factors that determine the magnitude of immunological functions in vitro and in vivo...we must be prepared to reevaluate one of the central dogmas concerning the role of GSH. GSH was viewed mostly as an important anti-oxidant that protects cells against oxidative stress" [178].

So the findings of Best et al [174], where cyanobacterial LPS markedly reduced GSTs in fish embryos after 24 hour exposures, may indicate an anti-inflammatory response displaying a different time course to that seen with heterotrophic bacterial LPS, or a consequence of decreased GSH availability, as intracellular GSH efflux increases with the onset of apoptosis [179]. GSTs can be inhibited by GSH conjugates, i.e. their reaction products [191], so this possibility may also need to be considered. In any event, and insofar as innate immune responses in fish embryos can be equated to mammalian cellular activities, these responses are likely to be very complex, with numerous endogenous mediators, feedback loops and time-dependent variation. As an example, the chemoprotecant oltipraz, which is thought to exert anticarcinogenic effects through induction of cytochrome P450 (CYP) enzymes, is now known to transiently (within 24 hours) inhibit some important CYPs and GSTs *in vitro *and *in vivo*. Animals treated for three days showed two-fold-plus increases in the same enzymes [192,193]. We would be interested to see time-course studies that replicate the work of Best et al [174], perhaps continued over several days.

In addition to the studies cited above, nine reports describe the isolation and purification of cyanobacterial LPS for various biomedical, biochemical or structural studies, but no toxicological endpoints were investigated [28,157,194-200]. The topic of cyanobacterial LPS was reviewed by Weckesser et al [27], and Mayer & Weckesser [201]. These reviews are especially valuable in that they contrast the work done on other photosynthetic prokaryotes, especially the purple non-sulfur bacteria, some of which have been shown to have lipid A structures that are LPS antagonists, as discussed above. No cyanobacterial lipid A structures have been described to date.

In addition to work done on isolated and purified LPS, some reports discuss the activity of cyanobacterial LPS by use of the *Limulus *amoebocyte (LAL) assay. This is a highly sensitive test, though there are trade-offs in terms of specificity, with other bacterial products such as peptidoglycan and glucan capable of registering positive responses [49,202,203]. The assay may not always be a reliable predictor of cellular and *in vivo *responses [204]. Seydel et al [205] suggest that, while the LAL assay is useful for detecting and quantifying LPS in blood products, it is not a measure of endotoxicity. The study of Rapala et al [206] had some significant findings pertaining to the topic of presumed toxicity of cyanobacterial LPS. The authors used the LAL assay to test 26 axenic strains from five cyanobacterial genera; all responses were at least five orders of magnitude lower than reactions to *E. coli *LPS, and several were below the assay's detection limits. This suggests that the lipid A structures of these LPS have some significant and fundamental structural differences to endotoxic lipid A, as the LAL assay does not react to some unusual or modified lipid A structures [88].

### Spirulina platensis: the importance of exposure route

*S. platensis *has a long history of use as a foodstuff, dietary supplement and livestock feed additive, with its probable use dating back to the ninth century in Africa, and the 14^th ^century in Mexico [207]. *Spirulina *is classified taxonomically under the genus *Arthrospira*, order Oscillatoriales; *A. maxima *and *A. fusiformis *are grown commercially in mass culture, but usually designated as "*S. platensis*" [208]. The use of this cyanobacterium was comprehensively reviewed by Ciferri [209], who concluded that extensive nutritional and toxicological testing has shown it to be a safe and valuable protein source. The use of *Spirulina *was briefly reviewed along with that of other edible microalgae by Kay [42], who cited Ciferri [209] in stating that some "negative effects" of *Spirulina *feeding were seen in multigenerational studies and mutagenicity tests. However, this appears to be a misinterpretation on the part of Kay [42], as Ciferri [209] described "negative results" from these studies. The original publications cited by Ciferri [209] were unobtainable. More recent studies seem to support the suggestion that consumption of *Spirulina *is not harmful, and enhances various immune functions [210-214].

A single case report is the exception to the rest of the literature. Iwasa et al [215] describe a 52-year-old male taking antihypertensive, hypolipidaemic and hypoglycaemic pharmacotherapy, who developed abnormal hepatic enzyme levels two weeks after taking *Spirulina*, presumably on a regular basis. Liver function tests showed a significant deterioration over the following three weeks, after which he was hospitalised. Although a physical examination was unremarkable, a liver biopsy revealed some degenerative changes. Serological studies for a range of viruses were negative. His medications and *Spirulina *were withdrawn, after which his hepatic function rapidly returned to normal. Hepatotoxicity in this case was attributed to consumption of *Spirulina*, on the basis of temporal relationships between liver function abnormality and recovery with consumption and withdrawal of *Spirulina*, although possible interaction effects with the medications would have been worth considering. This may have been the case with simvastatin, the cholesterol-lowering agent this individual was taking. Simvastatin causes increased proinflammatory cytokine production, and it can potentiate inflammatory responses induced by bacterial products [216]. A brief anecdotal report described two separate occurrences of gastro-intestinal illness in adults following consumption of *Spirulina *pills in Canada in the early 1980s, though there was an indication that one individual's tablets had some bacterial contamination [217].

Recent work has demonstrated that *A. fusiformis *in Kenyan soda lakes is capable of producing the cyanobacterial exotoxins microcystin-YR and anatoxin-a [218,219]. The implications of this finding are important because cyanobacterial poisoning is implicated in mass mortalities of Lesser Flamingos in the Rift Valley, and *A. fusiformis *is the principal food source for these animals [220,221]. Common toxigenic cyanobacteria such as *Anabaena *and *Microcystis *are also found in these lakes, and periodically dominate the phytoplankton profile [222], so presumably toxin-producing genes are being transferred between these genera in the field.

The long-standing and widespread consumption of *Arthrospira *spp. illuminates the importance of considering the route of exposure in toxicology studies, and the dangers in this case of presumptive inference of disease from the findings of parenterally administered LPS. Tornabene et al [223] reported a lethal dose of *Spirulina platensis *LPS in the range of 400 mg/kg (i.p. mouse), although those findings are not supported by the work of Stewart, where *Spirulina *LPS i.p. injections of 350 mg/kg failed to induce either thermoregulatory change or sickness behaviour signs [156]. However, Falconer [224] reported that cell lysates of *Spirulina *were highly toxic to mice when administered by intraperitoneal injection.

Other cyanobacteria are consumed as foods, medicines and dietary supplements. Wild-harvested *Aphanizomenon flos-aquae *was over a decade ago reportedly "consumed by thousands of people without incident" [42], although the lake that produces the commercially available product (Lake Klamath, Oregon) is sometimes subject to contaminating growth of *Microcystis *spp., and some *A. flos-aquae *end-product batches contaminated with microcystins have since been found [225-227]. A Canadian survey analysed for microcystins in cyanobacterial products (*Aphanizomenon*, *Spirulina *and unidentified cyanobacteria) but did not present the results according to the component genera, so it is not clear whether *Spirulina *products (presumably originating from commercial mass cultures) contained microcystins [228]. *Nostoc commune*, a terrestrial cyanobacterium, has a long history of use in China and Scandinavia as food and medicine [229]. The widespread use of these products serves as a reminder that some cyanobacteria, and therefore their LPS, are not harmful by the oral route.

### LPS by inhalation

In our opinion, the sole natural exposure route that might explain aquatic cyanobacterial LPS-related illness is via inhalation of aerosolised cells or fragments. Extrapolating from the understanding of Gram-negative bacterial LPS on the respiratory system (as have most if not all of the authors cited in Table 1 for the presumed involvement of cyanobacterial LPS on various disease states), there is a significant and increasing body of literature on the association between endotoxin and pulmonary disease, including asthma, chronic obstructive airway disease and emphysema. Intact bacteria and cell wall fragments are readily aerosolised; bioaerosols of Gram-negative bacteria are widespread contaminants of soils, water and living organisms [230,231]. Exposure to airborne endotoxin has been associated with a range of occupational respiratory diseases, in industries where high concentrations of organic dusts are liberated, e.g. various agricultural settings, cotton milling, brewing, waste processing [230,232]. Endotoxin is also found in high concentrations in air pollution and household dust [233]. Endotoxin in some aquatic environments can be aerosolised to disease-related concentrations: Rose et al [234] investigated outbreaks of granulomatous pneumonitis affecting lifeguards at an indoor swimming pool, with some affected chronically. Gram-negative bacteria, principally *Pseudomonas *spp., colonised water spray systems in the facility, and increased endotoxin in bio-aerosols was linked to the illnesses.

Michel [232] reviewed experimental inhalation studies of LPS: 4–12 hour periods of dyspnoea, chest tightness, myalgia, shivering, fatigue and malaise with or without fever were reported in a minority of normal subjects. Impaired pulmonary function in the form of bronchoconstriction, changes in non-specific bronchial hyperresponsiveness and reduced alveolar-capillary diffusion were demonstrated. Asthmatic subjects responded with significant bronchoconstriction lasting five or more hours at doses of 20 μg, whereas normal subjects required doses of 80 μg or more to produce moderate bronchoconstriction [232]. Of interest is the observation that LPS-induced lung changes are associated with neutrophil activation, whereas purified allergen extracts induce bronchial eosinophilia in asthmatic subjects [232,235]. Normal subjects exhibit a broad range of responses to inhaled LPS: 9% of subjects developed airway obstruction after low-dose inhalation, and 15% showed a negligible airway response to high doses of LPS [233,236]. Polymorphisms in genes coding for Toll-like receptors, especially Toll-like receptor-4, appear to be important determinants of variability in human responses to inhaled endotoxins. Arbour et al [237] showed that a TLR4 sequence mutation is associated with an endotoxin hyporesponsive phenotype in humans.

Schwartz [233] describes asthma as a complex, heterogeneous disease with multiple clinical sub-types, polygenic inheritance, and influenced by many different environmental exposures. Endotoxin is one such exposure, which causes a biologically unique form of asthma [233]. However, exposure to endotoxin early in life may confer beneficial effects: growing up on a farm and exposure to livestock is reportedly associated with a significant reduction in atopy, and there is an inverse correlation between house-dust endotoxin concentration and allergen sensitisation [238-240]. This so-called "hygiene hypothesis" for allergic diseases describes the concept that allergy results from an imbalance in the T-helper cell (Th) subset. According to this theory, exposure to bacterial and viral pathogens in the prenatal and early childhood periods prevents the induction of allergen-associated Th2 cells by establishing a Th1-biased immunity [238,241]. However, the hygiene hypothesis is complex and controversial, with contradictory observations and refinements to the theory appearing in the literature. Interested readers are directed to some recent reviews and updates: [241-244]

LPS and allergens initiate inflammatory processes in the airways through different pathways and cytokine cascades: LPS is recognised by innate immune cells, principally alveolar macrophages, which generate pro-inflammatory cytokines such as IL-1, TNF-α and IL-8; the latter cytokine recruits and activates neutrophils. LPS also generates IL-12, which inhibits IgE responses. Allergens generate IL-4, IL-13 and IL-5, the latter cytokine being an activator of eosinophils [241].

In the context of environmental exposures, endotoxins and allergens often occur together; synergistic effects are important considerations in that airway responses to combinations of LPS and allergen are reportedly greater than to either substance alone in atopic asthmatics [241,245].

The impact of cyanobacteria on respiratory symptoms in atopic individuals is worthy of investigation, and may involve protein allergens and cyanobacterial endotoxin from both toxic and non-toxic blooms. However, the relative burden of cyanobacterial endotoxin to respiratory morbidity will depend on the capacity of the LPS of any given cyanobacterial species to act as an LPS agonist, or as an LPS antagonist, or be biologically inactive; such properties are as yet largely undetermined.

An equally important research effort should be directed towards the capacity of inhaled cyanobacterial exotoxins to generate immunologically non-specific responses (i.e. in unsensitised individuals) in the bronchial tree. Microcystin-LR appears to be able to efficiently gain access to the circulation by both intranasal and intratracheal routes [246-248], but Gram-negative bacterial endotoxin delivered by inhalation does not cross into the pulmonary vasculature to enter the circulation, and at least one endotoxin-stimulated cytokine – TNF-α – is compartmentalised in the airways [241,249,250]. What is open to question is whether the serious cases of pneumonia reported after recreational exposure to cyanobacteria (see Stewart et al [251]) may be explained by the induction of an inflammatory response by inhaled cyanobacterial exotoxin, which progresses to recruitment and activation of neutrophils and is confined to the pulmonary alveolar compartment. The possibility is also open as to whether less dramatic reports of respiratory illness may also be explained by a similar, albeit self-limiting process, in healthy, non-atopic individuals. Of course, this speculation does not exclude the likelihood of different, overlapping mechanisms of disease that may explain these phenomena – protein allergens in some cyanobacteria may provoke symptoms in atopic individuals, such symptoms possibly being exacerbated by the presence of cyanobacterial and/or epiphytic bacterial endotoxins.

Cyanobacterial exotoxins may have the capacity to generate respiratory illness in non-atopic individuals, with endotoxins from cyanobacteria or commensal bacteria possibly augmenting the symptoms. The potential for cyanobacterial and/or contaminant endotoxin alone to produce symptoms by inhalation exposure remains open, given the observation that LPS can produce measurable airway function changes in animal models and in some healthy individuals [236,252-254]. Yet it remains unclear whether such experimentally-induced changes in the airway function of healthy volunteers correlate with symptoms of respiratory dysfunction.

## Conclusion

Lipid A, the endotoxic moiety of LPS, was in previous decades thought to remain constant across different Gram-negative bacteria [100]. This is now understood to be incorrect; many non-enteric bacteria are seen to vary in their lipid A structures. Because the biological activity of lipid A is determined by its structure, the toxic potential of non-enteric bacteria can vary. Gram-negative organisms occupying different ecological niches will not have the same requirements for growth, and their outer membranes can be expected to vary in order to meet different environmental conditions [31]. Endotoxic potential cannot be assumed to be lacking in the LPS of non-enteric bacteria, however, as seen in the high LPS agonist activity of lipid A from the non-pathogenic purple non-sulfur bacterium *Rubrivivax gelatinosus*, as discussed above. A similar example is given by another group of non-pathogenic bacteria, *Rhizobium *spp., the LPS from some of which are comparable to that of enterobacterial LPS in lethal toxicity and cytokine-inducing activity [255,256]. Determining the lipid A structures of various nuisance cyanobacteria would be an interesting exercise in itself, but regardless of the findings, proponents of the "cyanobacterial LPS is toxic" cause need to define plausible exposure routes to allow LPS to signal host receptors and initiate a pathogenic cytokine cascade.

From the discussion in this review, we will put the hypothesis that oral consumption of non-toxic cyanobacteria, i.e. absolutely or essentially free of any of the known cyanobacterial exotoxins, will not result in either vomiting or diarrhoea. This hypothesis would be falsified by experiments that show isolated cyanobacterial LPS or non-toxic crude extracts can cause gastrointestinal signs and/or pathology in a suitable model. Our impression is that reports of G-I symptoms in humans exposed to cyanobacterial products are indications of innate defences being signalled by exotoxins that have breached the intestinal barrier. Once this occurs, and gut permeability is sufficiently disrupted, LPS may well synergise the pathology of cyanotoxins, especially the hepatotoxins. From what little is known to date about the toxic potential of cyanobacterial LPS, i.e. that they are weakly toxic compared to those of the Enterobacteriaceae, gut-derived LPS would seem to be the more likely candidate for augmenting the pathology of cyanotoxins. *In vivo *studies of oral exposure to cyanotoxins would be well served by use of a vomiting-capable model, i.e. non-rodent experiments.

There does not appear to be good evidence that cyanobacterial LPS are likely to initiate cutaneous reactions in healthy people exposed in recreational or occupational settings. Cutaneous reactions to cyanobacteria are discussed in detail elsewhere [257-259].

Exposure to bio-aerosols containing cyanobacterial endotoxins may be worthy of investigation, but we are not convinced that cyanobacteria-related acute respiratory illness in non-atopic, non-allergic individuals is not equally or more likely to be explained by inhalation of cyanobacterial exotoxins. If some of the exotoxins turn out to possess ligands that stimulate innate immune responses (discussed further in [156]), then the large pool of resident alveolar macrophages would be prime candidates for involvement in respiratory defences. The outbreaks of bath-water fever in Scandinavia and Africa (see accompanying review by Stewart et al [251]) were, in our opinion, suspicious of involvement by cyanobacterial exotoxin breakthrough into reticulated supplies. Similar outbreaks in future should be vigorously investigated for cyanotoxins if there is a suggestion of significant cyanobacterial contamination of source water.

In conclusion, LPS of the Enterobacteriaceae are potent immunomodulatory and immunotoxic bacterial products that stimulate a wide variety of responses in mammals, not least of these being a desire to wax lyrical on the topic. Thus:

"Endotoxins possess an intrinsic fascination that is nothing less than fabulous. They seem to have been endowed by Nature with virtues and vices in the exact and glamorous proportions needed to render them irresistible to any investigator who comes to know them" [260].

And:

"The dual role of LPS as effector and target makes it a fascinating molecule which...still hides many miracles. It intrigues at the same time clinical, biological, chemical, and biophysical researchers..."[83].

Facetiousness aside, these workers are pointing out that there is much to learn about the LPS of the most widely studied Gram-negative bacteria, these being the Enterobacteriaceae. The understanding of cyanobacterial LPS is utterly miniscule by comparison, and we urge caution before continuing to attribute such a disparate range of symptoms in humans to contact with these materials without the required research evidence. Weckesser, Drews and Mayer wrote in 1979 that:

"...the picture obtained with the Enterobacteriaceae cannot be assigned to other Gram-negative bacteria without detailed investigations. Considering the broad spectrum in morphological and physiological diversity of the many taxonomic groups of both photosynthetic bacteria and cyanobacteria, there is a wide open field for studies on the composition of their cell wall." [27].

Ressom et al [261] stated that:

"Given the enormous heterogeneity in LPS from Gram-negative bacteria there is every reason to suspect that the same will apply to cyanobacterial LPS and, due to their taxonomic distance apart, cyanobacterial LPS are likely to be different from those found in Gram-negative bacteria."

We agree with these statements.

## Abbreviations

CYP cytochrome P450

DNA deoxyribonucleic acid

G-I gastrointestinal

GSH glutathione

GST glutathione *S*-transferase

IgE immunoglobulin E

IL interleukin

i.p. intra-peritoneal

LAL assay *Limulus *amoebocyte lysate assay

LBP lipopolysaccharide-binding protein

LD lethal dose

LPS lipopolysaccharide/s

SEs staphylococcal enterotoxins

Th cell T-helper cell

TLR Toll-like receptor

TNF-α tumour necrosis factor-alpha

## Competing interests

The author(s) declare that they have no competing interests.

## Authors' contributions

IS conducted the review; PJS and GRS supervised the work and contributed to redrafting the paper. All authors read and endorsed the final manuscript.
